# The prognostic value of over-expressed TrkB in solid tumors: a systematic review and meta-analysis

**DOI:** 10.18632/oncotarget.19561

**Published:** 2017-07-25

**Authors:** Chunze Zhang, Xiaoting Li, Dan Gao, Haihua Ruan, Zhenzhen Lin, Xiaobo Li, Guang Liu, Zhicheng Ma, Xichuan Li

**Affiliations:** ^1^ Department of Immunology, Tianjin Medical University, Tianjin, China; ^2^ Tianjin Union Medical Center, Tianjin, China; ^3^ Department of Chemistry and Chemical Biology, University of New Mexico, Albuquerque, New Mexico, United States; ^4^ Tianjin Key Laboratory of Food Science and Biotechnology, College of Biotechnology and Food Science, Tianjin University of Commerce, Tianjin, China; ^5^ Department of Gastrointestinal Surgery, Tianjin First Center Hospital, Tianjin, China

**Keywords:** solid tumors, anoikis, TrkB, prognosis, meta-analysis

## Abstract

It is reported recently Tropomyosin-related receptor Kinase B (TrkB) plays key roles in the anoikis resistance during the processes of tumorigenesis and metastasis. However, its prognostic significance for clinical patients remains inconclusive. In order to establish a correct and practicable link between increased TrkB and prognostication of human solid tumors, a meta-analysis was performed in this article. A systematic literature research in the electronic databases PubMed, Embase and Web of Science was performed to identify eligible studies. A fixed-effects meta-analytical model was employed to correlate TrkB expression with OS, DFS and clinicopathological features. A total of 11 studies covering 1516 patients with various solid tumors were recruited in this meta-analysis. TrkB over-expression was associated with poorer OS and poorer DFS in multivariate analysis. Additionally, the pooled odds ratios (ORs) indicated that TrkB over-expression was associated with large tumor size, lymph node metastasis, distant metastasis and a higher clinical stage. Overall, these results indicated that TrkB over-expression in patients with solid tumors might be related to poor prognosis and serve as a potential predictive marker of poor clinicopathological prognosis factor.

## INTRODUCTION

Anoikis is a form of programmed cell death that occurs in anchorage-dependent cells when they detach from the surrounding extracellular matrix [1–3]. Anoikis acts as an important defense for the organism by preventing detached cells’ re-adhesion to new matrices in incorrect locations and their dysplastic growth [4]. Failure to execute the anoikis program and resistance to anoikis evolve as a hallmark of metastatic cancers which enables cancer cells to disseminate to distant organs through systemic circulation [5–7].

Tropomyosin-related receptor Kinase B (TrkB) is a tyrosine kinase receptor for brain-derived neutrophic factor (BDNF), which triggers several intracellular signals [8–10]. TrkB was originally defined as a specific suppressor of caspase-associated anoikis of non-malignant epithelial cells. High expression of TrkB promoted tumorigenesis and metastasis. The vast bulk of the evidence from studies on solid tumor cells of liver, lung, breast and ovary indicated that anoikis suppression induced by over-expressed TrkB best owed enhanced metastatic capacity on various tumor cells [11–14]. Therefore, TrkB is expected to be a potential drug target [15, 16]. the correlation between the expression of TrkB and patient survival remains inconclusive. Therefore, it is necessary to analyze the data of TrkB systematically in human solid tumors to draw a reasonable conclusion about its prognostic significance.

In this study, we conducted a meta-analysis to investigate the significance of over-expressed TrkB in the prediction of prognosis of corresponding patients. The results showed that increased TrkB expression in patients with solid tumors related to poor prognosis and served as a potential prediction of poor clinicopathological prognosis factor.

## RESULTS

### Study search information

**Figure 1:**
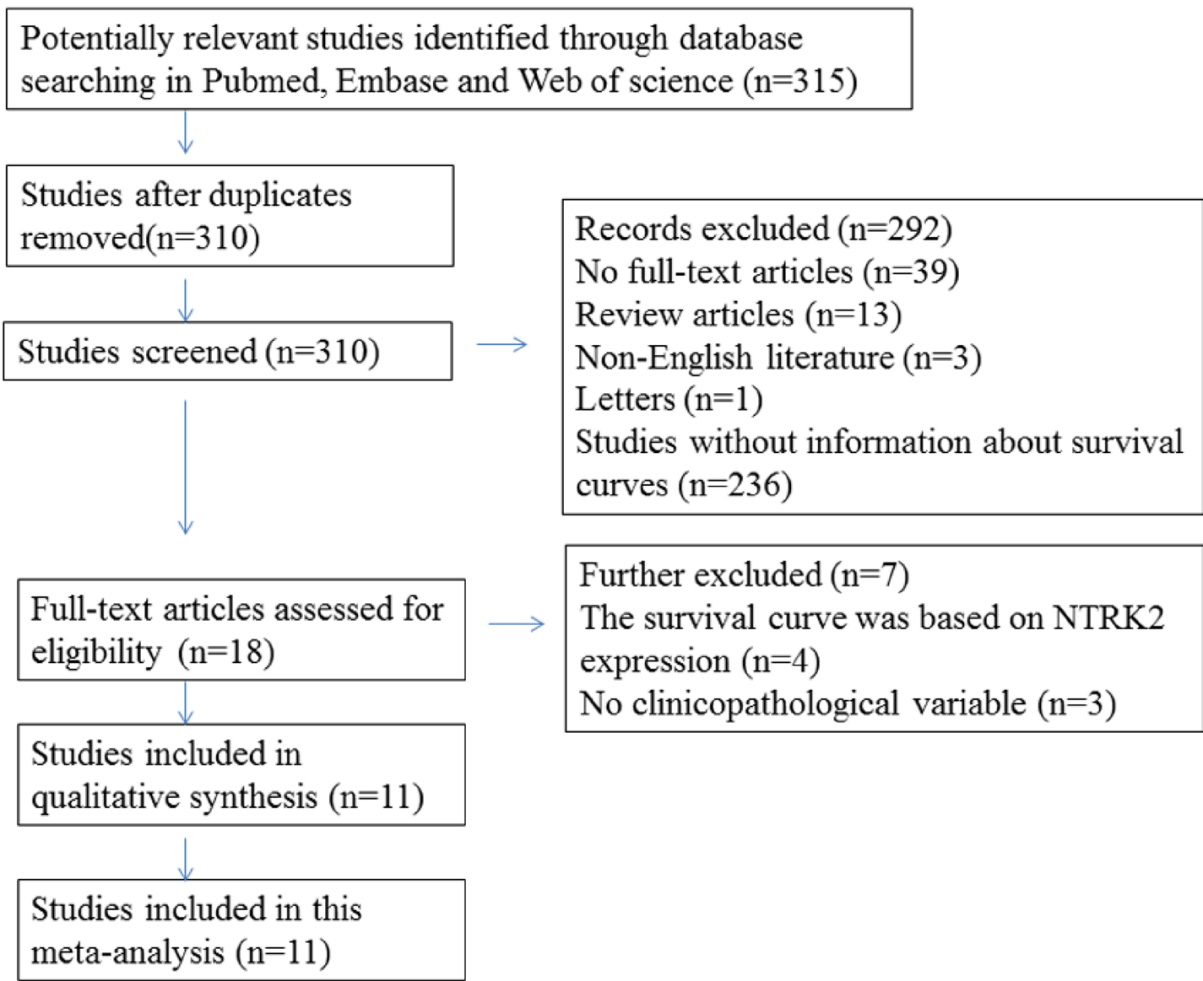
Flow diagram of the selection of eligible studies

The initial search identified 315 publications, of which, 18 studies were of acceptable relevance. However, four of these studies were excluded because the survival curve was based on NTRK2 expression, and three were excluded because of the absence of information about clinicopathological characteristics. Ultimately, 11 studies met the eligibility criteria and were included in the current meta-analysis (Figure 1).

### Description of the studies

The characteristics of the 11 identified studies were shown in Table 1. In total, 1516 patients from three regions (China, Greece and Japan) with 8 distinct cancers (gastric cancers [17–19], colorectal cancers [20, 21], nasopharyngeal carcinoma [22], non-small cell lung cancers [23], ovarian cancers [24], oral squamous cell carcinoma [25], hepaticellular carcinoma [26], sinonasal squamous cell carcinoma [27]) were included in these studies.

**Table 1:** Main characteristics of studies exploring the relationship between TrkB expression and tumor prognosis

Author	Year	Region	Cancer Type	Stage / Grade	No. of Patients	Age Median (Range)	Follow-up Time Median (range)	Detection Method	Staining position	Cut-off	Outcomes	NOS Score
Tanaka K [16]	2014	Japan	GC	pT1-pT4	320	68 y (18–90)	25.9 m (1.4–124.5)	IHC	Both nucleus and cytoplasm	> 10%	OS	8
Fan M [19]	2014	China	CRC	I–IV	191	NR	NR	IHC	No specific description	> 10%	OS	8
Li SS [21]	2013	China	NPC	I–IV	108	56 y (19–86)	60 m	IHC	Cytoplasm	> 10%	DFS, OS	7
Okugawa Y [17]	2013	Japan	GC	I–IV	150	NR	NR	IHC	Both nucleus and cytoplasm	> 10%	OS	8
Okamura K [22]	2012	Japan	NSCLC	I–IV	102	66.3 ± 10.19 y	1470 d	IHC	Membrane and cytoplasm	> 10%	DFS, OS	7
Sasahira T [24]	2013	Japan	OSCC	I–IV	102	68.7 y (48–79)	NR	IHC	Cytoplasm	> 10%	DFS	7
Au CW [23]	2009	China	OC	I–IV	94	20–83	NR	IHC	Membrane and cytoplasm	> 10%	DFS, OS	6
Zhang Y [18]	2008	Japan	GC	pT1-pT4	161	63 y (34–100)	NR	IHC	Cytoplasm	> 10%	DFS, OS	7
Dawson H [20]	2015	Greece	CRC	pT1-pT4	211	70.5 y (35–93)	NR	IHC	Membrane and cytoplasm	Membrane (+)	OS	8
Lam CT [25]	2011	China	HCC	I–IV	50	55 y (30–82)	NR	IHC	Membrane and cytoplasm	> 10%	OS	6
Li L [26]	2016	China	SSCC	I–IV	27	55 y (34–74)	NR	IHC	Membrane and cytoplasm	> 10%	DFS, OS	5

GC: Gastric Cancers; CRC: Colorectal Cancers; NPC: Nasopharyngeal Carcinoma; NSCLC: Non-Small Cell Lung Cancers; OC: Ovarian Cancers; OSCC: Oral Squamous Cell Carcinoma; HCC: Hepatocellular Carcinoma; SSCC: Sinonasal Squamous Cell Carcinoma; NR: Not Reported; OS: Overall Survival; DFS: Disease-Free Survival.

### Correlations of TrkB expression and OS

**Figure 2:**
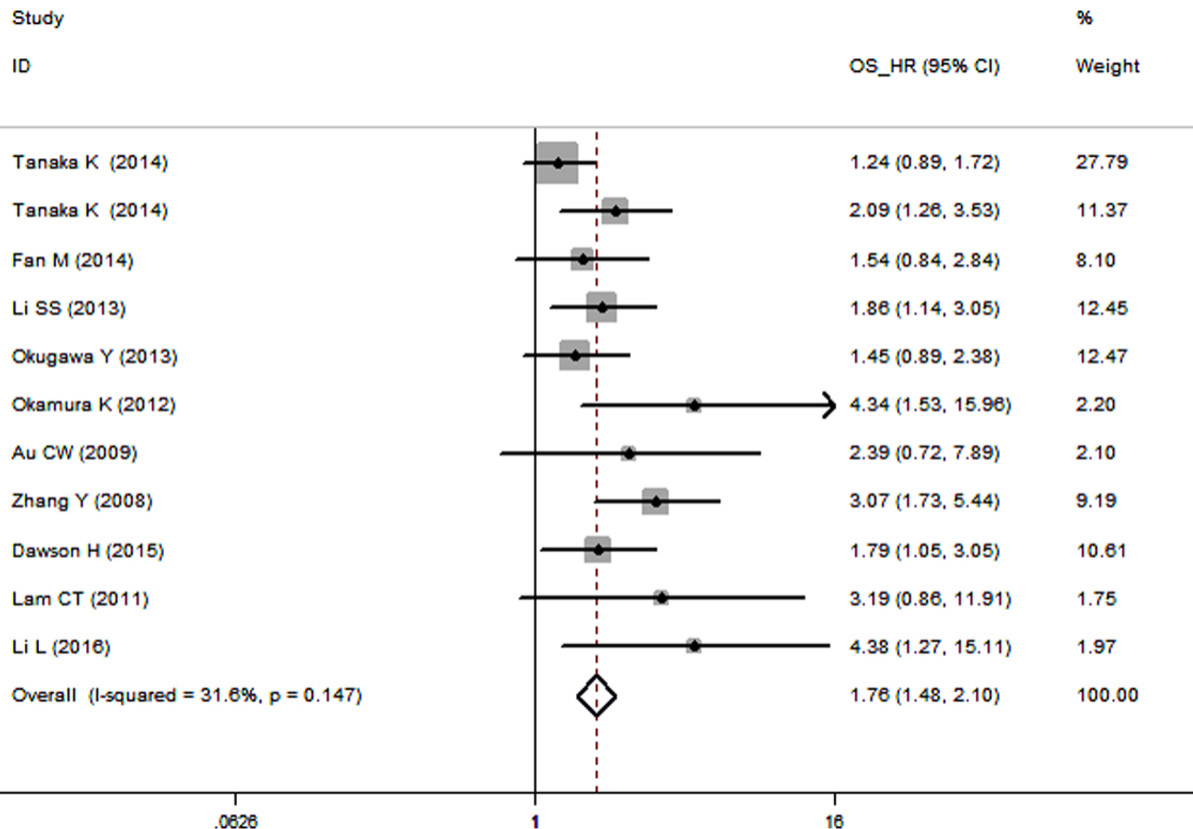
Forest plot describing the association between over-expressed TrkB and OS

The pooled hazard ratio (HR) revealed that over-expressed TrkB was significantly associated with poor overall survival (OS) for cancer victims in multivariate analysis (HR: 1.76, 95% CI: 1.48–2.10; Figure 2). However, no significant heterogeneity (*I*^2^ = 31.6%, *P* = 0.147) was observed when using a fixed-effects model to analyze the pooled HR of the OSs.

### Correlations of TrkB expression and DFS

**Figure 3:**
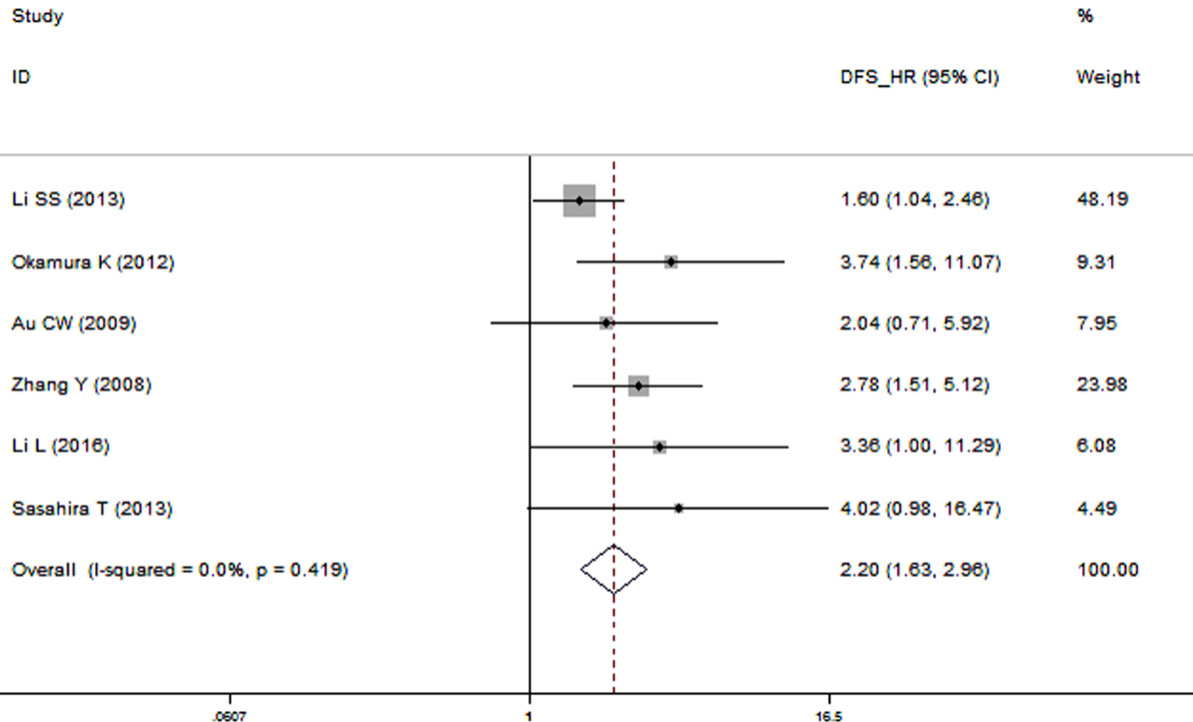
Forest plot describing the association between over-expressed TrkB and DFS

A significant correlation between over-expressed TrkB and disease-free survival (DFS) was also observed in the patients with solid tumors in multivariate analysis (HR: 2.20, 95% CI: 1.63–2.96; Figure 3) in the fixed-effects model without significant heterogeneity (*I*^2^ = 0.0%, *P* = 0.419).

### Correlations of TrkB expression and clinicopathological parameters

The clinical and pathological parameters collected from the eligible studies were presented in Table 2. Meanwhile, pooled results of the correlations were identified between over-expressed TrkB and clinicopathological features of patients with solid tumors. No significant correlation of over-expressed TrkB with gender and tumor differentiation were observed. However, the expression of TrkB was positively associated with large tumor size (OR: 1.96, 95% CI: 1.47–2.61), lymph node metastasis (OR: 1.93, 95% CI: 1.50–2.48), distant metastasis (OR: 2.76, 95% CI: 1.78–4.26) and a higher clinical stage (OR: 1.78, 95% CI: 1.16–2.72) in the fixed-effects model without significant heterogeneities (see Table 3).

**Table 2:** Summarized data of clinical and pathological parameters from the eligible studies

First author		Gender				Tumor differentiation				Tumor size				Lymph node metastasis				Distant metastasis				Clinical stage			
		Male		Female		Poor+Moderate/ undifferentiated		Well/differentiated		T3–4		T1–2		Yes		No		Yes		No		III-IV		I-II	
	TrkB+	-	+	-	+	-	+	-	+	-	+	-	+	-	+	-	+	-	+	-	+	-	+	-	
Tanaka K [16]		50	181	17	72	24	143	43	110	47	129	20	124	43	119	24	134	6	11	61	242	37	94	30	159
Fan M [19]		49	52	56	34	66	57	39	29	NA	NA	NA	NA	52	21	53	65	18	6	87	80	40	32	65	54
Li SS [21]		31	56	12	9	39	61	4	4	24	21	19	44	38	43	5	22	7	5	36	60	42	52	1	13
Okugawa Y [17]		54	65	10	21	25	50	39	36	46	52	18	34	50	55	14	31	6	6	58	80	47	55	17	31
Okamura K [22]		54	18	23	7	56	15	13	9	9	4	68	21	26	6	51	19	NA	NA	NA	NA	16	4	61	21
Sasahira T [24]		21	47	11	23	11	27	21	43	19	32	13	38	17	30	15	40	NA	NA	NA	NA	23	42	9	28
Zhang Y [18]		43	67	21	30	20	51	44	46	27	22	37	75	27	34	37	63	14	3	50	94	NA	NA	NA	NA
Dawson H [20]		27	41	26	46	48	74	4	13	44	71	8	16	31	48	21	39	11	8	42	78	NA	NA	NA	NA
Li L [26]		13	7	5	2	15	3	3	6	14	4	4	5	6	1	12	8	NA	NA	NA	NA	16	4	2	5

**Table 3:** Meta-analysis results of the associations of increased TrkB expression with clinicopathological parameters

Clinicopathological parameter	Ref	Overall OR (95% CI)	*P*-value	Heterogeneity test (*Q*, *I*^2^, *P*-value)
Gender (male vs female)	[16–22, 24, 26]	0.907 (0.703–1.169)	0.451	8.52, 6.1%, 0.384
Differentiation (poor VS well)	[16–22, 24, 26]	0.867 (0.524–1.435)	0.451	25.11, 68.1%, 0.3677
Tumor size (T3–4 vs T1–2)	[16–18, 20–22, 24, 26]	1.960 (1.473–2.608)	0.000	5.83, 0.0%, 0.559
Lymph node metastasis (yes vs no)	[16–22, 24, 26]	1.927 (1.499–2.477)	0.000	7.51, 0.0%, 0.483
Distant metastasis (yes vs no)	[16–21]	2.755 (1.781–4.260)	0.000	4.70, 0.0%, 0.453
Clinical stage (III-IV vs I-II)	[16–17, 19, 21–22, 24, 26]	1.775 (1.159–2.716)	0.008	9.52, 37.0%, 0.146

### Publication bias

**Figure 4:**
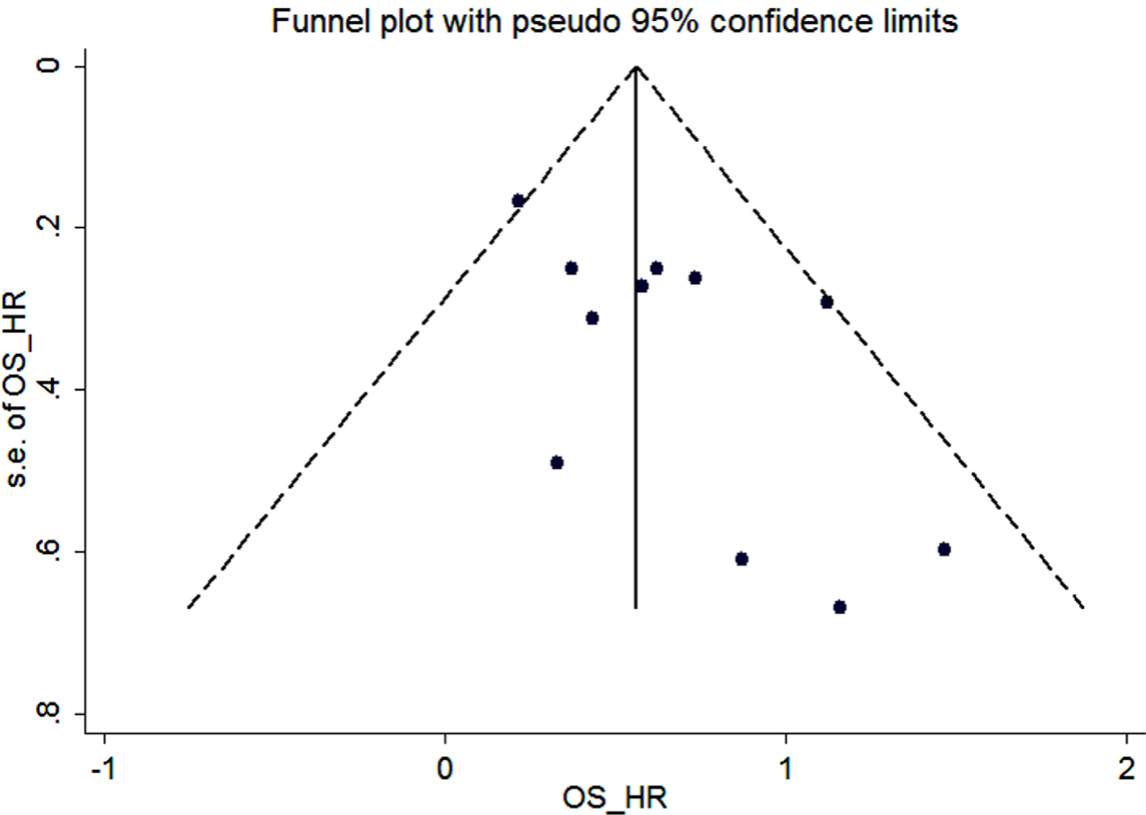
Funnel plot for the assessment of potential publication bias regarding OS in the meta-analysis

**Figure 5:**
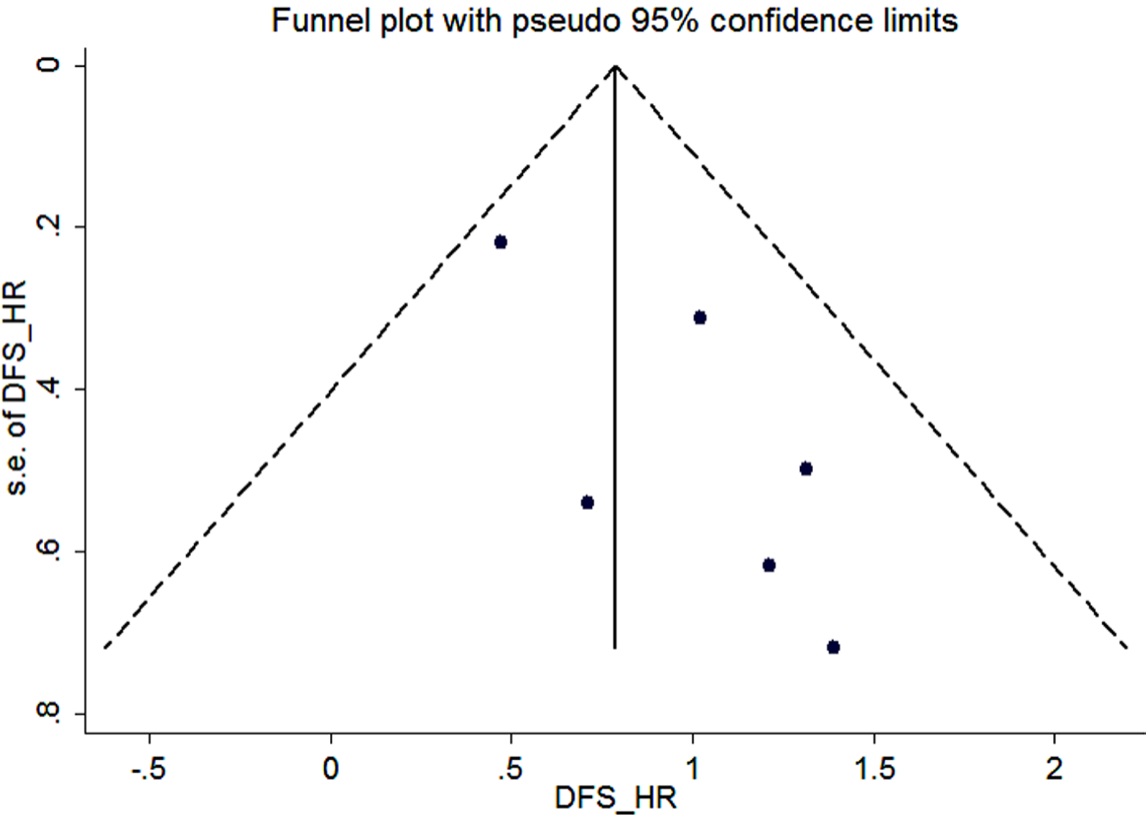
Funnel plot for the assessment of potential publication bias regarding DFS in the meta-analysis

We constructed funnel plots and performed Begg’s test to assess publication bias. As a result, the shape of the funnel plot for the OS, DFS and clinicopathological parameters seemed symmetrical in the multivariate analysis method (Figure 4, Figure 5 and Supplementary Figure 1).

## DISCUSSION

TrkB is a tyrosine kinase receptor for brain-derived neutrophic factor (BDNF), which triggers several intracellular signals. TrkB is over-expressed in various human malignancies and growing evidence demonstrated its association with tumour cell proliferation, invasion and metastasis [28–31]. TrkB is also an anoikis resistance related gene, whose over-expression may enhance a resistance of detachment-induced apoptosis. Consequently, knockdown of TrkB provided inhibition of growth or invasion and decrease of apoptosis in oral squamous cell carcinoma (OSCC) and endometrial carcinoma cells [25, 32]. However, thus far, no meta-analyses have been performed to evaluate the prognostic value of TrkB in cancer victims. This meta-analysis systematically estimates the association between TrkB expression and prognostic value of solid tumors.

In this meta-analysis, we evaluated survival data from 1516 solid tumor patients from 11 eligible studies, which met the inclusion criteria, were organized according to OS and DFS. For all studies, TrkB expression was detected by IHC. By meta-analysis of the 11 studies, we identified the pool HRs which indicated that TrkB was a factor in poor prognosis in various cancers. Because there is no significant heterogeneity among our included studies, so we did not perform further subgroup analyses. Additionally, no publication bias was observed. These results indicated over-expressed TrkB was positive related to poor OS and DFS in solid tumor patients. In addition, pooled results of the correlations were identified between over-expressed TrkB and clinicopathological features of patients with solid tumors. The results showed the expression of TrkB was positively associated with large tumor size, lymph node metastasis, distant metastasis and a higher clinical stage. We can explain this result by TrkB’s ability to enhance anoikis resistance, promote tumour cell proliferation, invasion and metastasis. Because of its involvement in these processes, TrkB is likely to be causally involved in tumor progression and, consequently, increased levels of TrkB would be expected to indicate a poor prognosis.

This meta-analysis was properly conducted, however, further analysis with several limitations would be considered in the future. first, need more trials to analysis; second, some of the survival data were extracted from Kaplan-Meier curves and might be less reliable than a direct analysis of variance; third, we need to search more non-English publications. In addition, the possible existence of unpublished studies could also result in potential publication bias. In general, concerning these limitations mentioned above, we must interpret these results with adequate caution.

In conclusion, our meta-analysis indicated that over-expressed TrkB was positive related to poor OS and DFS in solid tumor patients. Over-expressed TrkB could be served as a potential marker for poor clinicopathological prognostic factors in patients with various solid tumors. However, additional studies related to specific tumor types are necessary to illuminate the clinical utility of increased TrkB in solid tumors.

## MATERIALS AND METHODS

### Literature search strategy

This systematic review and meta-analysis is reported in accordance with the Preferred Reporting Items for Systematic Review and Meta-Analysis (PRISMA) Statement. The literatures relevant to TrkB expression and survival in solid tumors were searched in the PubMed, Embase and Web of Science databases through March 31, 2017. The search terms included the following key words in various combinations: TrkB, NTRK2, prognosis, prognostic, survival, and overall survival. The hits were limited to human studies and those published in English. The references list of review and bibliographies were further sifted to identify additional potentially relevant studies to avoid omission due to the electronic search approach.

### Study inclusion and exclusion criteria

The collected studies included in this meta-analysis had to meet the following criteria: (1) a pathological diagnosis of cancer was made; (2) TrkB expression in patients with any type of tumor was measured via immunohistochemistry; (3) associations of TrkB expression with OS, DFS or clinicopathological features were described; (4) HRs and 95% confidence intervals (CIs) were reported or could be calculated (based on the information in the paper); and (5) when the same author reported repeated results from the same population, the most complete report was included. The exclusion criteria for this meta-analysis were as follows: (1) unpublished papers; (2) laboratory articles, reviews and letters; (3) non-English language articles; (4) overlapping articles or ones with duplicate data; (5) articles with only animal experiments; (6) studies without information about survival curves; and (7) TrkB expression in patients with any type of tumor was analyzed only using RT-PCR method.

### Data extraction and quality assessment

All data were extracted independently by two investigators (Chunze Zhang and Dan Gao). For each eligible study, the following characteristics were extracted: first author’s name, publication year, region, type of cancer, number of patients, patients’ ages, follow-up times, detection methods, staining position, cut-off values, survival data (including OS and DFS) and clinicopathological parameters, such as tumor differentiation, tumor size, lymph node metastasis and TNM stage. For studies that presented only Kaplan-Meier curves was used to extract the survival data. The Newcastle-Ottawa-Scale (NOS) was adopted to assess the study quality of each individual study. The NOS score ranged from 0 to 9, and studies with NOS score ≥ 7 were defined as high-quality studies.

### Statistical analysis

This meta-analysis was performed using Stata 12.0 (Stata Corporation, College Station, TX, USA) software. Pooled estimates of HRs and their 95% CIs were used to estimate the association between TrkB expression and patients’ survival. The chisquared test (Cochrane’ s *Q* test) and I-squared statistical test were used to analyze the heterogeneity between studies. When the result of a *Q*-test (*I*^2^ > 50% or *P* < 0.05) indicated heterogeneity, the random-effects model was used for the meta-analysis. Otherwise, a fixed-effects model was used. HR with its value over 1.0 indicated poor prognosis patients with increased TrkB expression. Funnel plots were used to graphically represent the publication bias. Begg’s (rank correlation) test was adopted to confirm the publication bias.

## SUPPLEMENTARY MATERIALS FIGURE


