# Supplementary material for: The prognostic value of over-expressed TrkB in solid tumors: a systematic review and meta-analysis
Source: Oncotarget. 2017 Jul 25;8(59):99394–401. doi: 10.18632/oncotarget.19561 (PMC5725101; doi:10.18632/oncotarget.19561)
Supplement: Supplementary file 1 [file oncotarget-08-99394-s001.pdf]

The prognostic value of over-expressed TrkB in solid tumors: a systematic review and meta-analysis

SUPPLEMENTARY MATERIALS

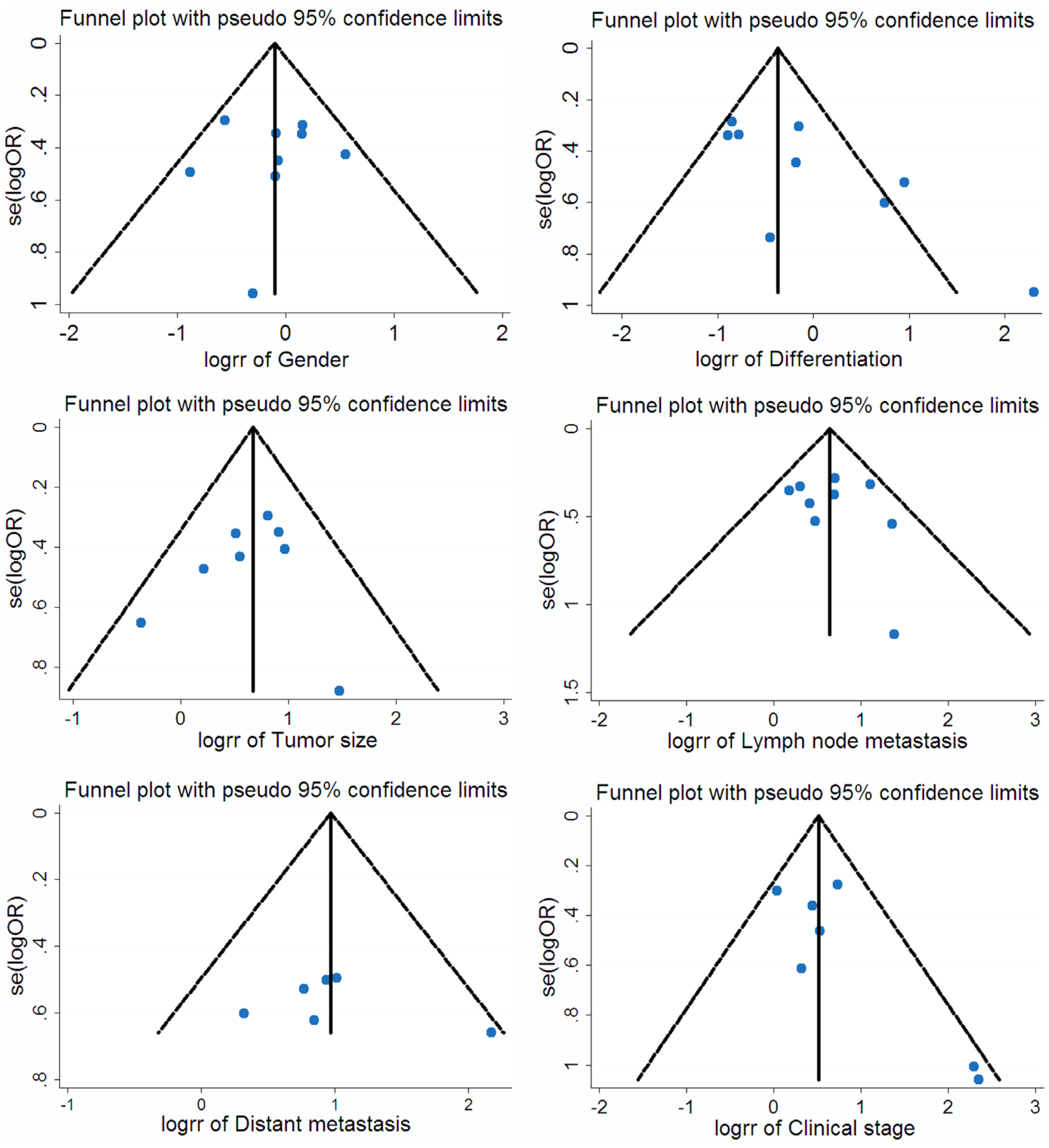

Supplementary Figure 1: Funnel plot for the assessment of potential publication bias regarding clinicopathological parameters in the meta-analysis.
